# Noninvasive Follicular Thyroid Neoplasm With Papillary-Like Nuclear Features Arising in the Thyroglossal Duct: A Case Report and Literature Review

**DOI:** 10.7759/cureus.93884

**Published:** 2025-10-05

**Authors:** Manami Momii, Shingo Umemoto, Takenao Tei, Takashi Hirano

**Affiliations:** 1 Department of Otorhinolaryngology, Head and Neck Surgery, Faculty of Medicine, Oita University, Yufu, JPN; 2 Department of Otolaryngology, Japan Community Healthcare Organization (JCHO) Nankai Medical Center, Saiki, JPN

**Keywords:** fine-needle aspiration cytology, noninvasive follicular thyroid neoplasm with papillary-like nuclear features, sistrunk procedure, thyroglossal duct cyst, thyroid cancer

## Abstract

A noninvasive follicular thyroid neoplasm with papillary-like nuclear features (NIFTP) is a recently reclassified thyroid tumor characterized by low malignant potential and typically confined to the thyroid gland. Its occurrence in a thyroglossal duct remnant is exceedingly rare, with only a few cases reported. Herein, we describe the case of a 74-year-old man who presented with a painless, progressively enlarging midline anterior neck mass. Ultrasonography and contrast-enhanced computed tomography demonstrated a well-circumscribed cystic lesion along the thyroglossal duct tract. Fine-needle aspiration cytology revealed a follicular-patterned lesion, consistent with Bethesda category IV. The patient subsequently underwent surgical excision via the Sistrunk procedure.

Histopathological examination showed a completely encapsulated follicular lesion with papillary-like nuclear features, without true papillae, capsular, or vascular invasion, thereby confirming the diagnosis of NIFTP arising in ectopic thyroid tissue. Given the absence of invasive features, no additional therapy was required, and no recurrence was observed at the six-month follow-up.

This case highlights the diagnostic limitations of preoperative cytology in ectopic thyroid lesions and underscores the importance of complete surgical excision when malignancy cannot be definitively excluded.

## Introduction

Ectopic thyroid tissue may develop anywhere along the embryologic pathway of the thyroid descent. The thyroglossal duct cyst (TGDC) represents the most common congenital neck anomaly, and in rare cases, may give rise to thyroid carcinoma, predominantly of the papillary subtype [1]. The typical differential diagnoses for midline neck masses include TGDC, dermoid cysts, epidermoid cysts, ectopic thyroid tissue, and lymphadenopathy. In adults, a solid or complex midline neck lesion warrants careful evaluation to exclude malignancy. Noninvasive follicular thyroid neoplasm with papillary-like nuclear features (NIFTP), a recently reclassified thyroid tumor entity, is characterized by follicular architecture, papillary‑like nuclear features, and noninvasive behavior [2,3]. Its occurrence within a thyroglossal duct is exceedingly rare and poses diagnostic challenges [4,5].

## Case presentation

History of present illness

A 74-year-old male presented with a painless anterior midline neck mass that had progressively enlarged over the past year. The mass was mobile during deglutition and tongue protrusion. Examination of the laryngopharynx revealed no abnormalities, and thyroid function tests were within normal limits.

Imaging and cytological findings

**Figure 1:**
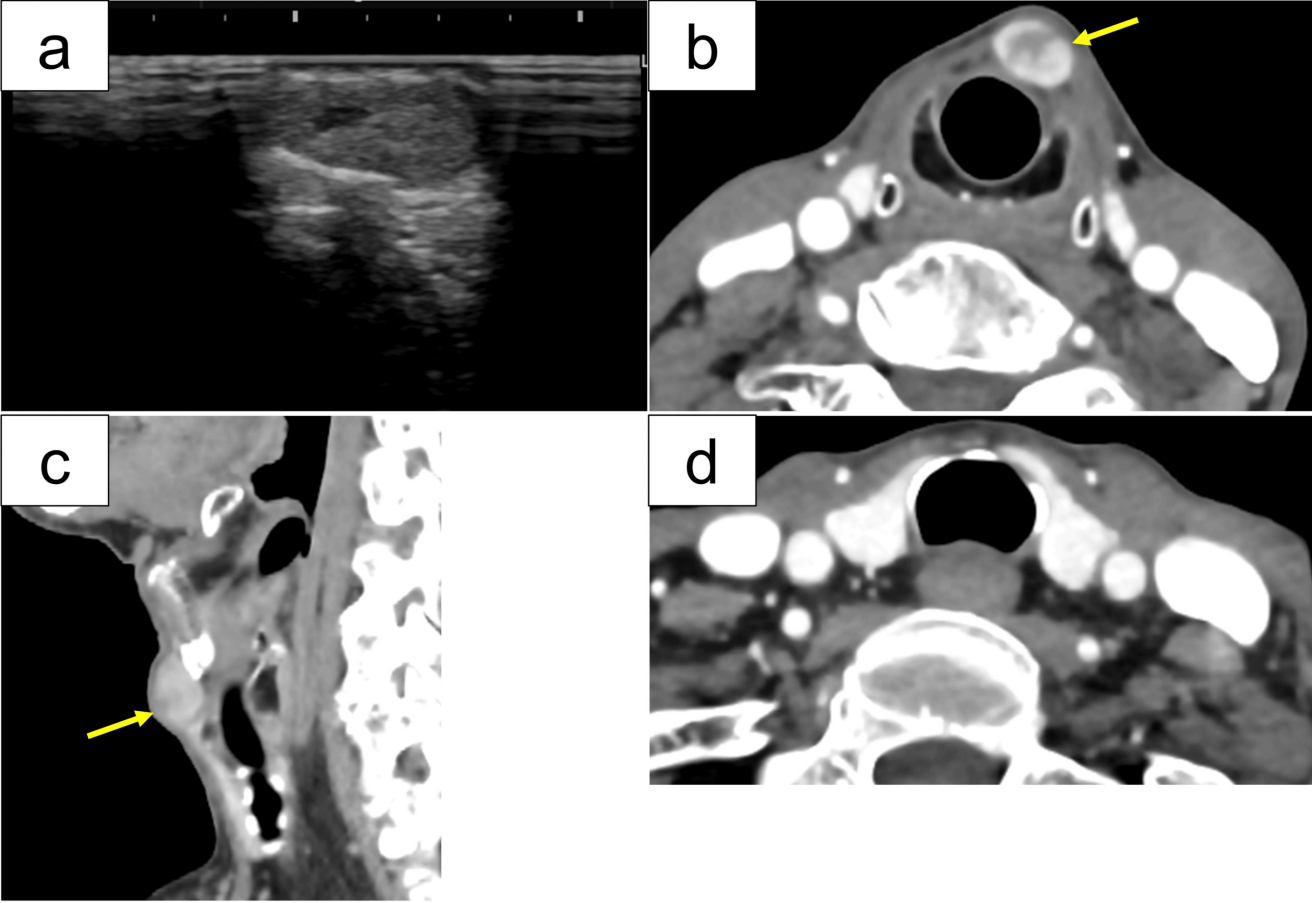
Preoperative imaging findings (a) Ultrasonography reveals a well-defined hypoechoic lesion with solid components and no internal calcification. (b, c) Axial (b) and sagittal (c) computed tomography (CT) images show a 10 × 10 mm mass located just superior to the thyroid cartilage with solid content (arrow). (d) Additional axial CT image confirming that the orthotopic thyroid gland is in the normal position without abnormalities.

Neck ultrasonography revealed a midline hypoechoic lesion with solid components, distinct from the orthotopic thyroid gland, and without internal calcification (Figure 1a). Computed tomography (CT) was performed to assess the extent of the lesion and its relationship to adjacent structures. CT showed a well-defined 10 × 10 mm mass located just superior to the thyroid cartilage, containing solid internal content but no calcification (Figures 1b, 1c). The orthotopic thyroid gland was in its normal position and appeared unremarkable (Figure 1d). Fine-needle aspiration cytology (FNAC) suggested a follicular neoplasm, Bethesda category IV.

Based on the midline location and imaging features, preoperative differential diagnoses included a thyroglossal duct cyst with mural nodule, ectopic thyroid neoplasm, epidermoid cyst, and, less likely, papillary carcinoma arising in the thyroglossal duct.

Surgical findings

**Figure 2:**
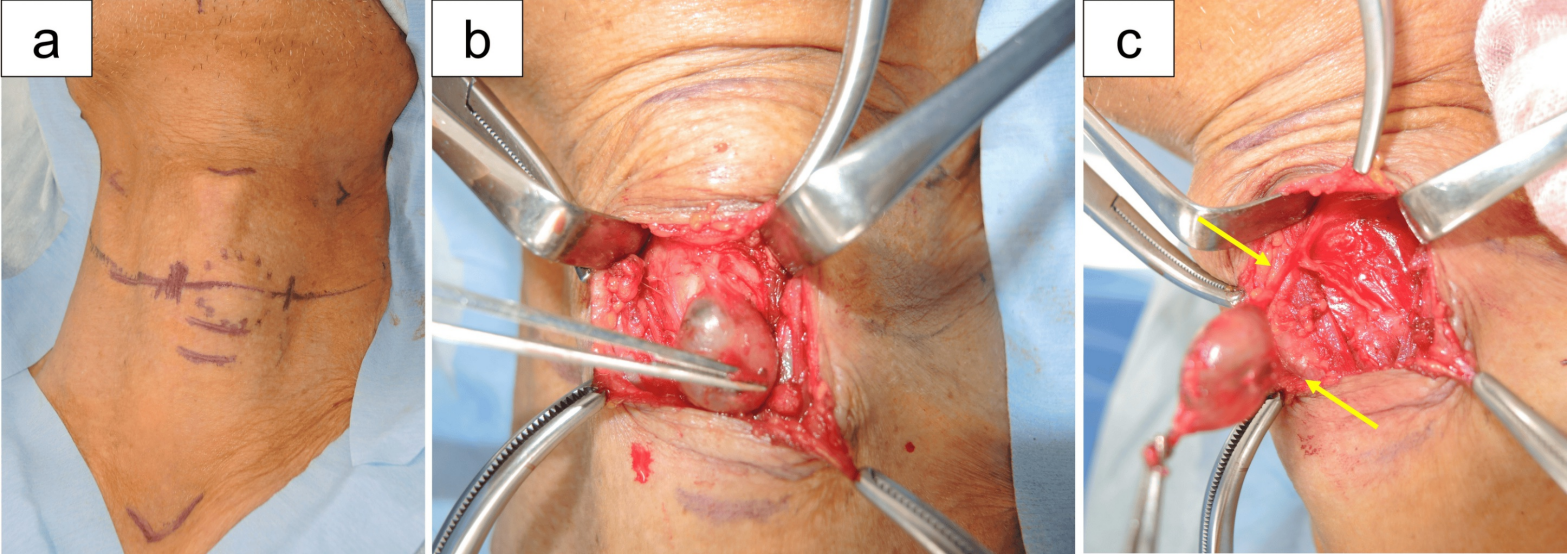
Intraoperative findings (Sistrunk procedure) (a) Skin incision at the level of the hyoid bone. (b) Subplatysmal flaps are raised to expose the strap muscles. The tumor was well-encapsulated and could be easily dissected from the surrounding tissues. (c) The encapsulated tumor is excised en bloc along with cord-like structures extending superiorly and inferiorly (arrows).

Given the inability to exclude malignancy, the patient underwent a Sistrunk procedure for both diagnostic and therapeutic purposes (Figures 2a-2c). Intraoperatively, the tumor was well-encapsulated and easily dissected from surrounding tissues, with no evidence of local invasion (Figure 2b). Additionally, the tumor was continuous with cord-like fibrous structures extending both superiorly and inferiorly (Figure 2c). The inferior cord was connected to the pyramidal lobe of the orthotopic thyroid gland. Both cords were excised en bloc along with the tumor. Grossly, the resected mass was well-circumscribed, smoothly surfaced, and elastic-hard in consistency.

Pathological findings

**Figure 3:**
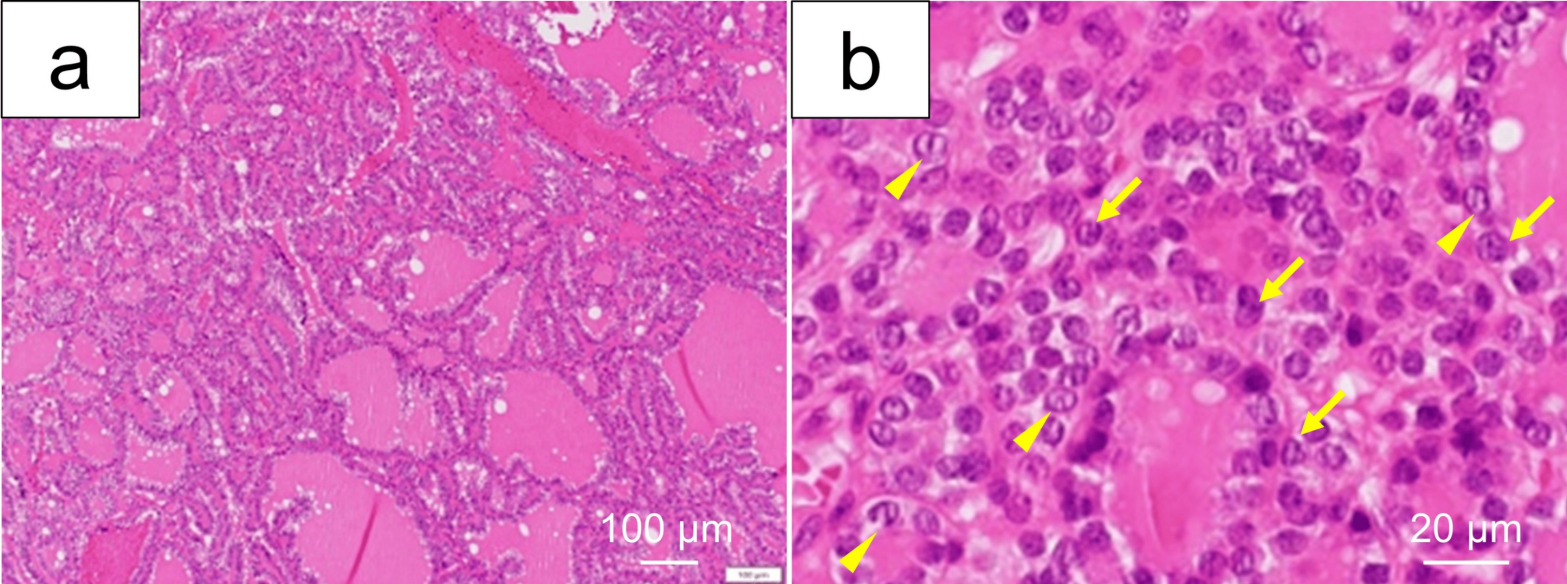
Histopathological findings of the thyroglossal duct lesion (hematoxylin and eosin staining) (a) H&E, ×40: Low-power view showing nuclear enlargement. Scale bar: 100 μm. (b) H&E, ×200: High-power view showing nuclear grooves (arrowheads) and chromatin clearing, consistent with papillary-like nuclear features (arrows). Scale bar: 20 μm. No capsular or vascular invasion was identified in any of the sections.

Histopathological examination revealed a follicular growth pattern with clear demarcation (Figure 3a). Nuclear enlargement, nuclear grooves, and chromatin clearing consistent with papillary-like nuclear features were observed (Figure 3b). No capsular or vascular invasion was identified in any sections. These findings were diagnostic of NIFTP.

Postoperative course

**Figure 4:**
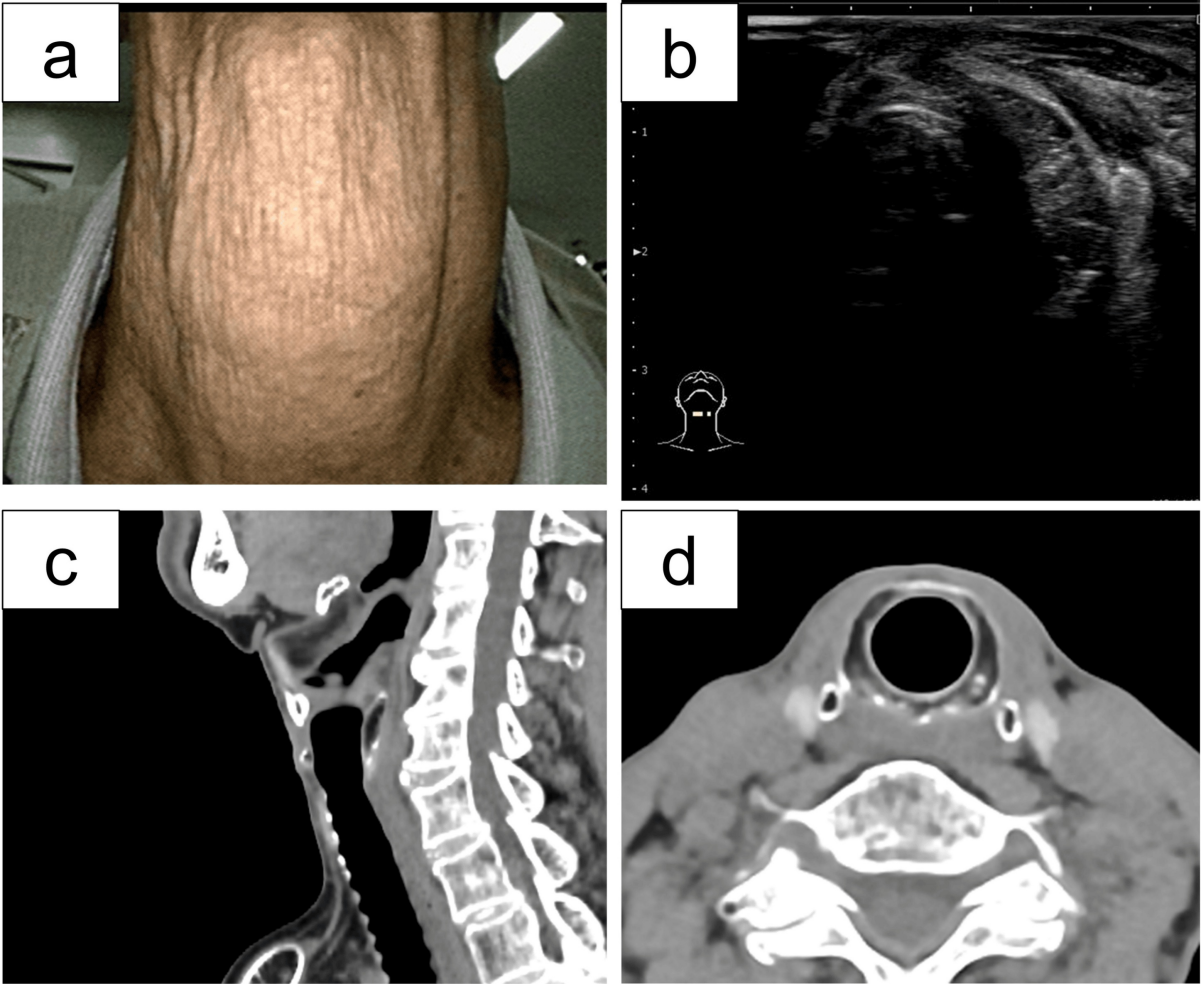
Postoperative clinical course (a) Local findings. Examination of the surgical site revealed no swelling, erythema, tenderness, or other abnormalities. (b) Ultrasonographic findings. Postoperative ultrasound of the anterior neck revealed no evidence of residual lesion or recurrence. (c, d) Computed tomography (CT) findings. Sagittal (c) and axial (d) CT images demonstrated no signs of local recurrence.

The postoperative recovery was uneventful. The patient was discharged on postoperative day 7 and followed regularly in the outpatient clinic without complications. At six months, no evidence of recurrence was observed (Figures 4a-4c), and the orthotopic thyroid gland remained normal (Figure 4d).

## Discussion

In this case, preoperative diagnosis was challenging because the lesion’s benign appearance on imaging contrasted with an indeterminate cytological result (Bethesda category IV). Although the lesion was clinically consistent with a benign midline neck mass, such as a thyroglossal duct cyst (TGDC), the possibility of malignancy could not be excluded. Therefore, surgical excision via the Sistrunk procedure was performed both for treatment and to establish a definitive diagnosis. Histopathological evaluation subsequently confirmed noninvasive follicular thyroid neoplasm with papillary-like nuclear features (NIFTP). This case highlights the diagnostic difficulty of detecting rare thyroid neoplasms in ectopic locations and emphasizes the importance of surgical management in such ambiguous presentations.

In the following sections, we discuss the key aspects of diagnosis, pathology, and management in greater detail, based on our case and the current literature.

Diagnostic challenges

NIFTP arising in the thyroglossal duct, particularly within lesions presumed to be TGDCs, is rare and often misdiagnosed preoperatively as a benign cystic lesion. Imaging findings are nonspecific, and FNAC is limited by sampling error and overlapping cytologic features. Sampling limitations and interpretive overlap with benign entities, such as thyroiditis or hyperplasia, have been cited as the primary reasons for misdiagnosis [6]. In the present case, FNAC categorized the lesion as Bethesda category IV, prompting surgical excision. Similar diagnostic approaches have been described in previous reports [4,7]. Additionally, distinguishing NIFTP from follicular variant papillary thyroid carcinoma (FVPTC) remains a critical challenge with significant prognostic and therapeutic implications [2].

Pathologic features

NIFTP is characterized by encapsulation, a predominantly follicular growth pattern, and the absence of true papillary structures or invasion. In most cases, these features are often sufficient to reach a diagnosis based on routine hematoxylin and eosin staining alone, particularly when the lesion is completely excised and histologically well-demarcated, as in the present case. Accordingly, neither immunohistochemical staining nor molecular analysis was required to establish the diagnosis.

In support of such diagnostic sufficiency, several key histopathologic distinctions between NIFTP and FVPTC have been well-documented. NIFTP typically demonstrates complete encapsulation, a purely follicular architecture, and no true papillae. Its nuclear features resemble those of papillary thyroid carcinoma but are generally less pronounced [8].

In contrast, FVPTC may exhibit either encapsulated or infiltrative growth, with more prominent nuclear atypia and occasional papillary structures [2,3]. Capsular and vascular invasion, which are diagnostic of FVPTC, are absent in NIFTP and remain critical in differential diagnosis [8-10]. On FNAC, NIFTP is most often classified as Bethesda category IV due to its follicular morphology, whereas FVPTC is more frequently assigned to categories V-VI, reflecting its papillary-like nuclear features [11].

Table 1 presents a detailed comparison of the histologic and cytologic characteristics of NIFTP and FVPTC.

**Table 1:** Cytologic-histologic comparison of NIFTP vs FVPTC NIFTP and FVPTC show overlapping but distinct pathologic features. The table was independently created by the authors by compiling data from the cited sources. NIFTP: noninvasive follicular thyroid neoplasm with papillary-like nuclear features; FVPTC: follicular variant papillary thyroid carcinoma.

Feature	NIFTP	FVPTC	Reference
Architecture	Follicular pattern, no true papillae	Follicular or mixed, may have true papillae	[2]
Nuclear features	PTC‑like (mild to moderate)	PTC‑like (often more prominent)	[8]
Encapsulation	Completely encapsulated	Encapsulated or infiltrative	[2,9]
Capsular/vascular invasion	Absent	May be present	[8]
Cytology (FNA)	Bethesda IV, limited specificity	Bethesda V–VI, more typical PTC findings	[11]

Molecular features

In cases where histologic features overlap with those of FVPTC or when tumor sampling is limited, further evaluation using immunohistochemical markers or molecular profiling may be required to distinguish NIFTP from malignant counterparts. For instance, RAS mutations (NRAS, HRAS, KRAS) are frequently detected in NIFTP, with reported frequencies ranging from approximately 46% to 59% in some cohorts [12]. However, these mutations are also common in FVPTC, limiting their specificity for preoperative distinction between the two entities [12-14].

Conversely, BRAF V600E mutations, while prevalent in classic papillary thyroid carcinoma (PTC), are rare or absent in NIFTP but may occur in FVPTC, particularly in infiltrative or aggressive subtypes [13]. TERT promoter mutations are also reported in approximately 10-15% of FVPTC cases but are generally absent in NIFTP [10]. RET/PTC rearrangements are also occasionally identified in FVPTC but are generally absent in NIFTP, providing further distinction [8,15]. Together, these molecular differences support the utility of genomic profiling when histologic diagnosis is inconclusive or when assessing the malignant potential of a lesion (Table 2).

**Table 2:** Molecular profiles of NIFTP compared to FVPTC Molecular alterations differ significantly between NIFTP and FVPTC. The table was independently created by the authors by compiling data from the cited sources. NIFTP: noninvasive follicular thyroid neoplasm with papillary-like nuclear features; FVPTC: follicular variant papillary thyroid carcinoma

Mutation Type	NIFTP	FVPTC	References
RAS mutations (NRAS, HRAS, KRAS)	Common (46～59%)	Common	[12-14]
BRAF V600E	Rare or absent	Occasionally present, especially in infiltrative subtypes	[13]
TERT promoter mutations	Rare or absent	Present in ~10–15%	[10]
RET/PTC rearrangements	Rare or absent	Occasionally present	[8,15]

Cytological limitations and Bethesda category IV

FNAC is essential in the evaluation of thyroid nodules; however, its diagnostic performance is limited in NIFTP cases, particularly when occurring in ectopic locations. Bethesda category IV lesions show high rates of false-negative and indeterminate results, with NIFTP frequently misclassified as benign hyperplasia or follicular neoplasm [5,6]. Because of the considerable overlap in cytological features, definitive diagnosis requires histological confirmation. Core needle biopsy has been proposed as a complementary approach to enhance preoperative diagnostic accuracy, particularly for lesions in deep-seated or atypical locations such as the thyroglossal duct [16].

Emerging role of preoperative molecular testing

In recent years, preoperative molecular testing using genomic classifiers such as ThyroSeq or Afirma GSC has emerged as a valuable adjunct in the evaluation of indeterminate thyroid lesions, including those categorized as Bethesda category III or IV [17,18]. These tests can be performed on material obtained via FNAC and help refine the diagnostic accuracy prior to surgery.

Genomic testing offers improved risk stratification, allowing clinicians to distinguish between benign and malignant nodules with greater confidence. For instance, the identification of high-risk mutations (e.g., BRAF V600E, TERT promoter) may influence surgical planning by prompting more extensive surgery, such as total thyroidectomy, instead of conservative excision. Conversely, the absence of high-risk alterations in a lesion with indeterminate cytology may support observation or limited surgery.

Although not yet widely available or routinely adopted in all settings, these molecular platforms represent a promising tool for individualized treatment planning and are increasingly incorporated into clinical algorithms. In the context of thyroglossal duct lesions with atypical cytology, preoperative molecular analysis may assist in distinguishing benign TGDCs from rare neoplasms, such as NIFTP or FVPTC, potentially guiding more appropriate surgical intervention.

Surgical considerations: role of Sistrunk procedure

Although initially performed for suspected TGDC, the Sistrunk procedure involves complete excision of the cyst, the central portion of the hyoid bone, and a cuff of tissue surrounding the thyroglossal tract, thereby ensuring adequate resection margins. This procedure is critical for both therapeutic and diagnostic purposes, particularly in lesions where malignancy cannot be excluded. By providing wider tissue clearance, the procedure reduces recurrence rates and facilitates more reliable histopathologic evaluation [19]. If the preoperative FNAC had indicated a higher suspicion of malignancy (Bethesda category V or VI), total thyroidectomy might have been considered in addition to the Sistrunk procedure, depending on the patient’s overall risk profile and clinical context.

Clinical implications and management

The reclassification of NIFTP has prompted a paradigm shift in the management of thyroid tumors, enabling clinicians to avoid total thyroidectomy and radioactive iodine therapy in low-risk cases. Comparable principles apply to tumors arising in the thyroglossal duct, provided that the orthotopic thyroid gland remains unaffected and complete excision is achieved [1,20].

While the patient remains recurrence-free at six months postoperatively, continued long-term follow-up is warranted. Given the typically indolent course of NIFTP, clinical and ultrasonographic surveillance will be conducted at regular intervals in accordance with current guidelines for low-risk thyroid neoplasms. The short follow-up period represents a limitation of the present report and warrants cautious interpretation of recurrence risk.

Taken together, these findings emphasize the importance of comprehensive evaluation, including clinical, imaging, cytological, and pathological correlation, when assessing atypical midline neck lesions. An integrated view of the diagnostic process, including clinical impressions, imaging, cytology, histology, and molecular considerations, is provided in Table 3.

**Table 3:** Summary of diagnostic and histopathological findings The table was independently created by the authors from case records and cited references. Molecular testing was not performed in this case, but its role is discussed based on current literature.

Step	Modality	Key Findings	Interpretation
Clinical	Palpation	Midline neck mass above the thyroid	Consistent with TGDC
Imaging	Ultrasound	Hypoechoic solid mass, no calcification	Suspicious for neoplasm
Imaging	CT	Well-defined midline mass, no calcification, normal thyroid	TGDC vs. neoplasm
Cytology	FNAC	Follicular architecture, Bethesda IV	Follicular neoplasm
Surgery	Sistrunk procedure	Complete excision of the lesion	Therapeutic and diagnostic
Histology	H&E staining	Encapsulated, follicular growth, no papillae or invasion	Consistent with NIFTP
Molecular (not performed)	Literature-based recommendation	Molecular profiling (e.g., RAS mutation, BRAF V600E negative) may aid diagnosis	Not conducted in this case; discussed in the literature

To the best of our knowledge, no previously documented cases have specifically described NIFTP arising within the thyroglossal duct itself. This case may therefore represent the first recognized instance of such a presentation, emphasizing the importance of considering even rare thyroid neoplasms in the differential diagnosis of midline neck lesions.

## Conclusions

This case reports a rare occurrence of noninvasive follicular thyroid neoplasm with papillary‑like nuclear features (NIFTP) arising in the thyroglossal duct. Although NIFTP is most commonly found in the orthotopic thyroid, its presence in a midline neck mass highlights the need for careful consideration of thyroid neoplasms in the differential diagnosis of thyroglossal duct lesions. Because preoperative distinction between benign and malignant lesions can be difficult, complete surgical excision remains essential for definitive diagnosis and treatment when malignancy cannot be excluded.
